# A cross sectional survey of attitudes, awareness and uptake of the parental pertussis booster vaccine as part of a cocooning strategy, Victoria, Australia

**DOI:** 10.1186/1471-2458-13-676

**Published:** 2013-07-23

**Authors:** Ellen J Donnan, James E Fielding, Stacey L Rowe, Lucinda J Franklin, Hassan Vally

**Affiliations:** 1 Victorian Government Department of Health, 50 Lonsdale Street, Melbourne, Victoria 3000, Australia; 2 National Centre for Epidemiology and Population Health, College of Medicine and Biological Sciences, The Australian National University, Building 62, Cnr of Eggleston and Mills Roads, Canberra Australian Capital Territory 0200, Australia; 3 Victorian Infectious Disease Reference Laboratory, 14 Wreckyn Street, North Melbourne, Victoria 3051, Australia; 4 School of Public Health and Human Biosciences, La Trobe University, Melbourne, Victoria 3086, Australia

**Keywords:** Diphtheria-tetanus-acellular, Pertussis vaccines, Parents, Cross-sectional studies, Victoria

## Abstract

**Background:**

The Victorian Government Department of Health funded a diphtheria, tetanus and acellular pertussis vaccine for parents of infants from June 2009 to June 2012 as part of a cocooning strategy for the control of pertussis. The aim of this study was to assess parents’ attitudes and awareness of the vaccination program, and to estimate vaccine uptake.

**Methods:**

A cross-sectional survey of 253 families with a child born in the first quarter of 2010 residing within five metropolitan and four rural local government areas in Victoria was conducted. Univariate analyses were performed to describe the relationship between demographic variables, knowledge and awareness of the disease, the vaccine program and vaccine uptake. Multivariate analyses examining predictors for awareness of the vaccine program and for the uptake of vaccination were also conducted.

**Results:**

One hundred and five families were surveyed (response rate 43%). Of these, 93% indicated that they had heard of ‘pertussis’ or ‘whooping cough’ and 75% of mothers and 69% of fathers were aware the pertussis vaccine was available and funded for new parents. Overall, 70% of mothers and 53% of fathers were vaccinated following their child’s birth, with metropolitan fathers less likely to be vaccinated as rural fathers (RR = 0.6, p = 0.002). Being a younger mother (p = 0.02) or father (p = 0.047), and being an Australian-born father (RR = 1.9, p = 0.03) were found to predict uptake of the vaccine in parents.

**Conclusion:**

Parents indicated a reasonable level of knowledge of pertussis and a willingness to be vaccinated to protect their child. However, vaccine uptake estimates indicated further opportunity for program improvement. Future cocooning strategies would benefit from specifically targeting fathers and metropolitan maternity hospitals to increase vaccine uptake. Wider promotion of the availability of vaccine providers may increase uptake to maximise the success of cocooning programs. Further investigation of the effectiveness of the cocooning strategy in decreasing infant morbidity and mortality is required.

## Background

Victoria is a south-eastern state of Australia, which has a population of 5.6 million and comprises approximately 25% of the Australian population [1]. Between 2009 and 2011, epidemic numbers of pertussis were seen in Victoria, with 3,737 cases in 2009, 6,960 cases in 2010, and 8,812 cases in 2011 notified to the Department of Health [2]. This was a notable rise compared with the 1,673 cases notified in 2008 and 1,053 cases in 2007 [2,3]. Whilst pertussis affects people of all ages, infants less than six months of age have the greatest morbidity, as evidenced by the number of hospitalisations and deaths [4].

Peri-natal vaccination of close contacts of infants is known as cocooning. Close contacts may include mothers, fathers, grandparents and other household members. The primary objective of cocooning is to reduce disease transmission to infants from their closest contacts, whilst secondarily reducing morbidity from pertussis in adults [5]. A time-limited funded vaccination program for parents of infants was implemented by the Victorian Government Department of Health from 15 June 2009 in response to the rising incidence of pertussis [6]. The program provided adult diphtheria, tetanus and acellular pertussis vaccine [dTpa] free of charge to hospitals, general practitioners and local councils for administration to parents. The Department of Health actively promoted the program using circulars to immunisation providers, posters for doctors’ surgeries and Maternal and Child Health Centres, and distributed fact sheets for professional bodies (including Divisions of General Practice, General Practice Victoria and individual general practitioners), childcare centres, kindergartens and other stakeholders such as hospitals, Playgroup Victoria and Grandparents Victoria [7]. Funding for the program ceased on 30 June 2012.

This study aimed to assess parents’ knowledge of pertussis infection, their attitudes towards the free vaccination program, and to estimate uptake of the vaccine among eligible parents following the birth of their child. It also sought to examine factors predicting awareness of the vaccine program and uptake of the vaccine.

## Methods

### Study design and participant selection

**Figure 1:**
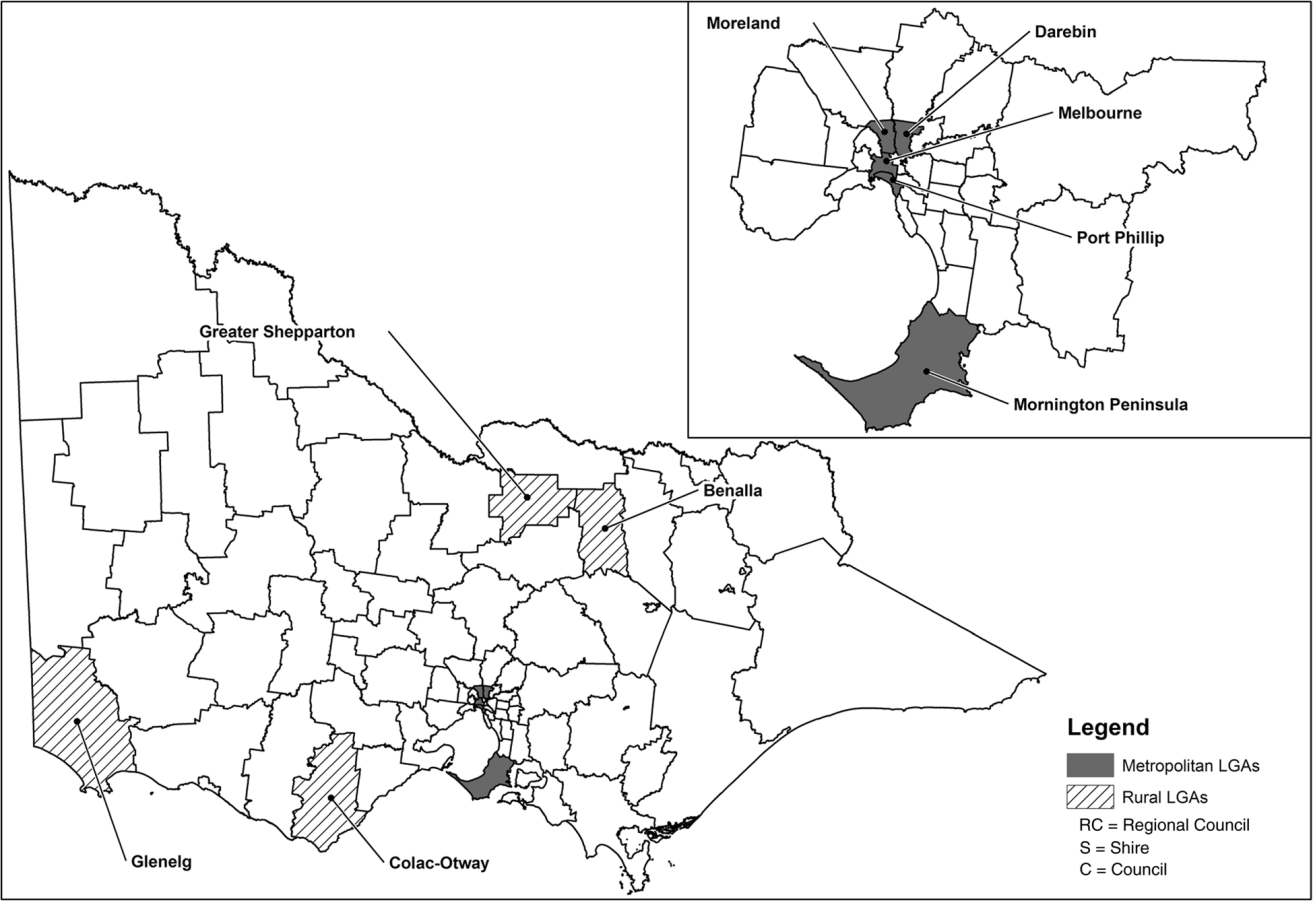
Victorian Local Government Areas (LGAs) that participated in the study.

A cross-sectional survey of parents of infants was designed to assess the attitudes, awareness and uptake of the pertussis vaccine for parents in Victoria. Victorian Local Government Areas (LGAs) were stratified into metropolitan and rural localities, and five LGAs were randomly selected from each stratum. Rural LGAs were oversampled due to a perception that vaccination would be less accessible outside the metropolitan areas. Five metropolitan LGAs and four rural LGAs participated in the study (Figure 1). A sample size of 270 was calculated for parental vaccination using 80% power, a significance level of 0.05, and an estimation of parental vaccine coverage based on vaccine distribution data which estimated approximately 70,000 vaccines distributed for a birth cohort of approximately 70,000 and 140,000 parents (assuming two parents per birth) giving an estimate of vaccine coverage of 50% (personal communication: Mr. Michael Batchelor, Manager, Immunisation Section, Department of Health, 8 March 2010). A Def factor of 1.5 was used to take into account the stratified sampling technique. A list of unique identifiers for each birth from each of the selected LGA’s Maternal and Child Health records was obtained for the period 1 January to 31 March 2010 and these records were computer randomised to select 30 families. Two hundred and fifty-three families who had a child born in the first quarter of 2010 were selected across the participating LGAs using this two-stage stratified sampling. Two rural LGAs did not have sufficient births registered in the three month study window, so the period was extended to 1 January to 30 April 2010 for these LGAs only. Contact details for the selected families were obtained from the participating LGA’s Maternal and Child Health records. One rural Maternal and Child Health program chose to telephone the selected families to request consent to post the questionnaire to them; 13 of the 30 families consented to participate. One metropolitan LGA did not provide parental contact details, instead directly mailed a generic letter, questionnaire and reply-paid envelope to the selected families. Families were sent an introductory letter, study questionnaire and a reply-paid envelope approximately six to eight months following the birth of their child in September/October 2010. A reminder letter and questionnaire was sent to families for whom a response had not been received three weeks after the initial mail out.

### Questionnaire design

A four page questionnaire was designed to assess parents’ awareness of pertussis and the cocooning program, their attitudes towards a cocooning strategy and uptake of the vaccination. The disease was referred to as both pertussis and whooping cough in the introductory letter; however the term whooping cough was used throughout the questionnaire. The questionnaire consisted of Likert scales to assess knowledge and attitudes, and closed questions to assess program awareness and vaccine uptake. Parents were also given the opportunity to comment on the program. Demographic details were collected on parents and the self-reported vaccination status for their child. Parents were asked about their knowledge of pertussis illness in adults and infants, the adult pertussis vaccine, whether parents had heard about the vaccination program, if they were vaccinated, and if not vaccinated their reasons for not being vaccinated.

### Data analysis

A study database was created in EpiInfo™ and data analyses were completed using Intercooled Stata™ Version 9.0. Percentages were calculated for most responses including questions utilising Likert scales. Maternal demographic data was compared to the most recent available data on births in Victoria [8]. The proportions of parents vaccinated were adjusted using the direct method of standardisation, and are based on the weighted sum of the LGA-specific proportion of parents vaccinated, using the 2009 Estimated Resident Population of each participating LGA as the standard population. Relative risks and associated confidence intervals were calculated, with a significance level of p < 0.05 used to assess predictors to program awareness and vaccine uptake. Multivariate analyses examining predictors for awareness of the vaccine program and uptake of the vaccine were conducted using backwards stepwise logistic regression using the likelihood-ratio test for variables statistically significant in univariate analysis.

### Ethics approval

Ethics approval to conduct this research was gained from the Australian National University Human Research Ethics Committee. The research was also approved by the Victorian Government Department of Education and Early Childhood Development’s Early Childhood Research Committee.

## Results

### Demographics

Of the 253 questionnaires mailed, 108 were completed and returned (response rate = 43%). Six were returned without being received by their intended recipients. Three families did not meet the inclusion criteria for the study, leaving 105 eligible families. Responses from these families represented 4-5% of registered births in participating metropolitan LGAs and 4-40% of the registered births in rural LGAs (Table 1).

**Table 1:** Survey responses and births registered in the study period by participating Local Government Area (LGA)

**LGA**	**Metropolitan/rural classification**	**Survey reponses (n = 105)**	**Registered births (n)**	**Proportion of births (%)**
Benalla	Rural	8	39	21
Colac-Otway	Rural	12	43	28
Darebin	Metropolitan	9	202	4
Glenelg	Rural	16	40	40
Melbourne	Metropolitan	12	226	5
Moreland	Metropolitan	10	279	4
Mornington Peninsula	Metropolitan	16	419	4
Port Phillip	Metropolitan	14	349	4
Shepparton	Rural	8	223	4

The median age of mothers was 33 years (range 20 to 41 years) and fathers was 34 years (range 22 to 59 years). Most families (92%) were either married or in a defacto heterosexual relationship and 8% were single mothers. Eighty-four percent of mothers and 78% of fathers were born in Australia; none identified as Aboriginal and/or Torres Strait Islander. A bachelor degree or higher was the most common highest level of educational attainment reported by mothers and fathers, with 46% and 36%, respectively, reporting having completed a tertiary degree. Twenty-seven percent of mothers and 34% of fathers reported having completed Year 11 or 12, with 22% and 20% having completed a diploma or certificate level education, and 6% and 10% having completed Year 10 or below.

Combined household income was slightly skewed to higher income households with 29% of respondents with a household income greater than (AUD) $95,000 and 22% between $65,000 and $95,000. One quarter of those surveyed reported a household income of $45,000 to $65,000, 11% reported an income of $25,000 to $45,000 and 13% of households earned less than $25,000. Almost all children from the families surveyed (98%) were born in hospital and 97% of families indicated that their child had either completed, or intended for their child to complete, the primary course of pertussis-containing vaccines as per the National Immunisation Program schedule. A comparison between mothers in this sample and the most recently available births data for Victoria (2008) showed that the sample roughly approximated the population for maternal age, marital status and place of birth, however there was a statistically significant difference between the proportions of mothers born in Australia between the survey sample and the Victorian population (Table 2).

**Table 2:** **Demographics of study participants compared to all Victorian women who gave birth in 2008 [**[8]**]**

**Demographic information**		**This study**	**Births in Victoria 2008**	**p-value**
Median maternal age (years)		33	31	ND^1^
Marital status (%)	Married/defacto	92	87	0.13
	Single	8	12	0.21
Maternal country of birth (%)	Australia	84	73	0.02
	Other	16	27	0.01
Place of birth (%)	Hospital	98	98	1.00
	Birthing centre	1	2	0.46
	Planned home births or unplanned out-of-hospital births	1	1	1.00

^1^ ND – Not done due to insufficient information available.

### Disease awareness and vaccine uptake

Ninety-three percent of parents indicated that they had heard of the disease whooping cough or pertussis. More than 80% of mothers and fathers agreed or strongly agreed that their infant was at risk of contracting pertussis, and 95% of mothers and 96% of fathers thought that pertussis infection could cause their child to be seriously ill. Thirteen percent of mothers and 17% of fathers did not agree that as an adult they were at risk of contracting pertussis (Table 3).

**Table 3:** Parents’ knowledge of pertussis and the adult vaccine

	**Parents**^**1,2**^	**Strongly disagree**	**Disagree**	**Neither agree or disagree**	**Agree**	**Strongly agree**
As an adult, I am at risk of contracting pertussis	Mother	6 (6%)	7 (7%)	20 (19%)	49 (47%)	22 (21%)
	Father	7 (8%)	10 (11%)	15 (16%)	41 (45%)	18 (20%)
Pertussis could cause me to become seriously ill	Mother	3 (3%)	9 (9%)	17 (16%)	50 (48%)	25 (24%)
	Father	3 (3%)	8 (9%)	14 (15%)	47 (52%)	19 (21%)
Pertussis could cause me to be moderately unwell with a prolonged cough	Mother	3 (3%)	3 (3%)	12 (12%)	60 (58%)	26 (25%)
	Father	2 (2%)	3 (3%)	18 (20%)	49 (54%)	19 (21%)
I consider the adult pertussis vaccine safe	Mother	6 (6%)	1 (1%)	20 (19%)	44 (42%)	33 (32%)
	Father	5 (5%)	2 (2%)	21 (23%)	40 (44%)	23 (25%)
The adult pertussis vaccine is effective in preventing pertussis	Mother	3 (3%)	4 (4%)	29 (28%)	47 (46%)	20 (19%)
	Father	2 (2%)	3 (3%)	27 (30%)	40 (45%)	17 (19%)
As a newborn, my child is, or was, at risk of contracting pertussis	Mother	6 (6%)	3 (3%)	9 (9%)	39 (38%)	46 (45%)
	Father	6 (7%)	5 (5%)	6 (7%)	35 (38%)	39 (43%)
Infection with pertussis could cause my child to be seriously ill	Mother	3 (3%)	0 (0%)	2 (2%)	30 (29%)	69 (66%)
	Father	3 (3%)	0 (0%)	1 (1%)	30 (33%)	57 (63%)

^1^ n Mothers = 104.

^2^ n Fathers = 91.

Seventy-five percent of mothers and 69% of fathers were aware that free (funded) pertussis vaccine was available for new parents. Mothers commonly heard about the vaccine from the Maternal and Child Health Nurse (43%) or their GP (13%). Friends, family, professional colleagues, immunisation staff, childcare, or a poster at a health centre were cited as other sources of information about the vaccine (13%). Fathers commonly heard about the program from their partner (35%), a Maternal and Child Health nurse (23%) or their GP (13%). Several factors were examined as predictors for awareness of the vaccine program, including parent’s age, country of birth, relationship status, household income, child’s place of birth (e.g. Hospital, birthing centre, home, other), and whether their child was vaccinated for pertussis, however no statistically significant predictors were found.

Seventy percent of mothers and 53% of fathers had received the pertussis vaccine following the birth of their most recent child. This equated to both parents being vaccinated in 56% of families (including single parents if they were vaccinated), one parent in 17% of families and none in 27% of families. Maternity hospitals were the most common place for mothers to receive vaccine (37%) followed by the local council (35%) and general practice (28%). Fathers were more likely to have the vaccine administered in general practice (46%) followed by maternity hospital (30%) and local council (23%). Not being aware of the availability of the free vaccine was the main reason reported for not being vaccinated (Table 4). No difference in relationship status, annual household income or highest educational level was found between vaccinated and unvaccinated parents. However, vaccinated mothers were on average of 2.7 years younger than unvaccinated mothers (p = 0.02) and vaccinated fathers were 3.1 years younger than unvaccinated fathers (p = 0.047). Australian-born fathers were 1.9 times (95% C.I. 1.0-3.8, p = 0.03) more likely to be vaccinated than overseas-born fathers. Both mothers and fathers who were aware of the vaccination program for new parents were 2.1 times more likely to be vaccinated than those that were not aware of the program (mothers: 95% C.I. 1.2-3.6, p = 0.0003; fathers: 95% C.I. 1.1-3.9, p = 0.004).

**Table 4:** Parents reasons for not being vaccinated*

**Reasons for not being vaccinated**	**Mothers n (%)**	**Fathers n (%)**
Already had the pertussis vaccine in last five years	2 (7%)	2 (5%)
Not aware of risks	3 (10%)	4 (9%)
Don’t consider the disease serious enough to be vaccinated	2 (7%)	1 (2%)
Not aware of the free vaccine	18 (62%)	18 (42%)
Costs associated with going to the doctor	2 (7%)	2 (5%)
Time and effort involved with getting vaccinated	2 (7%)	11 (26%)
Potential side effects of the vaccine	7 (24%)	7 (16%)
Concerns regarding how well the vaccine works	2 (7%)	4 (9%)
My religious beliefs	0 (0%)	1 (2%)
Other	3 (10%)	4 (9%)

*more than one reason could be identified.

There was a considerable difference in vaccination of mothers between metropolitan (64%) and rural residence (80%), however this difference was not statistically significant (p = 0.08). In contrast, the difference in vaccination levels of fathers between metropolitan (40%) and rural (73%) residence was statistically significant (p = 0.002). Fathers residing in metropolitan LGAs were less likely to be vaccinated than fathers in rural LGAs (RR 0.6, 95% CI 0.4–0.8). After adjustment for vaccine awareness, age and whether born in Australia or overseas, metropolitan fathers had 5.5 fold lower odds compared with rural fathers of being vaccinated (adjusted OR 0.2, 95% C.I. 0.1-0.6). Most parents residing in rural LGAs were vaccinated at their maternity hospital (71% of mothers, 46% of fathers). In the metropolitan LGAs, most mothers were vaccinated at local councils (62%) and the majority of fathers were vaccinated either at their general practitioner (48%) or at local councils (43%) (p < 0.001). The overall proportion of mothers vaccinated was 68%, and for fathers it was 49% when adjusting for population sizes of the contributing Victorian LGAs.

### Attitudes to parental vaccination and the cocooning strategy

Nearly all surveyed mothers and fathers (96%) indicated they would be prepared to be vaccinated to prevent transmitting an infectious disease to their child, with a general practice clinic the preferred location to receive the vaccine indicated by most mothers (65%) and fathers (70%). Parents were asked about their preferred place for vaccination: local council was preferred by 19% of mothers and 10% of fathers and maternity hospital by 15% of mothers and 19% of fathers. Parents indicated that knowledge about the disease, the level of risk to themselves or their child from the disease, how well the vaccine works, and potential side effects were important when making decisions regarding adult vaccination. The majority of parents gave low or no importance to the cost of the vaccine, the cost of going to the doctor to be vaccinated, the time and effort to be vaccinated and religious beliefs (Table 5).

**Table 5:** Factors affecting parents’ decisions to be vaccinated to protect their child

	**Parents**^**1,2**^	**Not at all important**	**Of low importance**	**Neutral or undecided**	**Moderately important**	**Very important**
How much I know about the disease	Mother	2 (2%)	8 (8%)	8 (8%)	40 (38%)	47 (45%)
	Father	2 (2%)	6 (7%)	10 (11%)	37 (41%)	36 (40%)
How at risk my child or I are from the disease	Mother	0 (0%)	1 (1%)	6 (6%)	11 (11%)	84 (82%)
	Father	0 (0%)	0 (0%)	7 (8%)	14 (16%)	69 (77%)
How well the vaccine works	Mother	0 (0%)	1 (1%)	14 (14%)	28 (28%)	59 (58%)
	Father	0 (0%)	3 (3%)	11 (12%)	25 (28%)	51 (57%)
Potential side effects of the vaccine	Mother	1 (1%)	2 (2%)	11 (11%)	28 (27%)	60 (59%)
	Father	1 (1%)	1 (1%)	13 (14%)	26 (29%)	49 (54%)
The cost of a vaccine	Mother	25 (24%)	23 (22%)	16 (16%)	24 (23%)	15 (15%)
	Father	18 (20%)	27 (30%)	14 (16%)	17 (19%)	13 (15%)
The cost of going to the doctor to be vaccinated	Mother	26 (25%)	24 (23%)	15 (15%)	23 (22%)	15 (15%)
	Father	19 (21%)	26 (29%)	12 (13%)	18 (20%)	14 (16%)
The time and effort to be vaccinated	Mother	32 (32%)	30 (29%)	16 (16%)	15 (15%)	9 (9%)
	Father	26 (29%)	23 (26%)	18 (20%)	13 (15%)	9 (10%)
My religious beliefs	Mother	85 (82%)	3 (3%)	10 (10%)	3 (3%)	2 (2%)
	Father	73 (82%)	4 (5%)	8 (9%)	1 (1%)	3 (3%)

^1^ n Mothers = 105.

^2^ n Fathers = 91.

## Discussion

The primary aim of this study was to assess parental attitudes towards, and awareness of, the funded parental pertussis vaccination program that was implemented by the Victorian Department of Health in 2009 as part of a cocooning strategy. Uptake of the vaccine by parents under this program was also estimated. Although the Victorian cocooning strategy for pertussis ended in 2012, the results of this study provide useful data that may be used to inform the implementation of similar programs in future. In particular, information on parents’ attitudes towards the use of parental vaccination as part of a cocooning strategy could be used to inform program materials for parents and to develop communication strategies for future programs. After adjustment for LGA populations, the proportion of mothers surveyed who received pertussis vaccine following the birth of their most recent child was 68%. For fathers, the proportion was 49%. These proportions were higher than expected given vaccine distribution records indicated that sufficient vaccine had only been distributed to cover 50% of new parents (Personal communication: Mr Michael Batchelor, Manager, Immunisation Section, Department of Health, 8 March 2010). It needs to be acknowledged that this finding may be indicative of the presence of selection bias in this study, with vaccinated parents possibly overrepresented in our sample. Of the parents who were not vaccinated, the most common reason given was a lack of awareness that a free vaccine was available. This suggests that additional funding to promote or advertise the vaccine to new parents may have resulted in greater uptake under the program implemented in 2009. In conducting these types of programs, funding allocated towards the development of a communications campaign aimed at promoting the program to parents, and considering incentives for hospitals, local councils, and general practitioners to inform and vaccinate their patients, may also assist with the uptake of vaccine.

In general, there was a high level of knowledge and awareness of pertussis as a childhood disease among parents in this study; 93% had heard of whooping cough or pertussis, 96% agreed or strongly agreed that pertussis could cause serious illness in infants, and 82% thought that infants were at risk of contracting pertussis. Although household contacts have been shown to be the most important source of infection for infants both in Australia [9,10] and worldwide [11], not all parents were aware that adults could contract and transmit pertussis to their child. This lack of awareness has been identified internationally as a barrier to vaccination, along with limited understanding of the potential for pertussis to cause severe disease in infants [12,13]. Consequently, educating parents of infants is an important step in removing barriers to vaccination; however, research has also shown that education alone is not sufficient to raise vaccination rates in parents [14].

The contrast between the numbers of parents residing in metropolitan areas who were vaccinated in hospitals compared with rural parents was striking. Only 6% of metropolitan mothers and 10% of metropolitan fathers were vaccinated in hospital, compared with 70% of rural mothers and 42% of rural fathers. The higher vaccine provision through rural hospitals could potentially be explained by differences in hospital policies between metropolitan and their (generally smaller) rural counterparts, particularly regarding the vaccination of fathers who would not be considered patients of the hospital. Maternity hospitals have been shown to be effective and timely providers of the pertussis vaccine to new parents, particularly amongst families of high risk infants such as neonates, with standing orders having been shown to successfully raise the vaccination rate to more than 80% of eligible women in the United States [15]. Further education and incentives (financial or otherwise) for maternity hospitals may increase uptake of post-partum mothers, particularly in metropolitan areas.

Whilst only 21% of mothers and 23% of fathers agreed that the time and effort to be vaccinated was important in their consideration of vaccination, the results indicated that this, in fact, was a common barrier for parents, particularly for fathers. Interestingly, some parents who indicated that they were not vaccinated due to time pressures responded that, in general, it was of little or no importance in their decision-making process. This suggests that, despite good intentions, the reality of life once a child is born can mean that time and effort are indeed important barriers to vaccination. This disconnection between intentions to be vaccinated versus actually obtaining a vaccination suggests that positive attitudes towards vaccination do not necessarily predict behaviour. Lack of time was found to be a major reason for not being vaccinated in a previous study where, despite an education program that demonstrated a significant increase in knowledge and willingness to be vaccinated for pertussis, only 8% of participants were vaccinated [13].

The major limitation in this study was the response rate of 43% which reduced the power of the study, and may have introduced some bias. A post-hoc power calculation estimated study power at 41.2%. One likely reason for the low response rate was due to the fact that parents of young children have multiple competing demands on their time and thus have limited time to respond to surveys. It is unclear as to whether those that responded to the survey were more likely to be vaccinated or had greater knowledge of pertussis but this possibility has to be considered. Unfortunately due to time and resource constraints, only one reminder letter was sent to parents requesting their participation in this study. Additional contact from the researchers may have increased participation, which may have minimised responder bias. Despite this, the comparison with the most recent report available on births in Victoria in 2008 [8] showed similar characteristics for maternal age, marital status and place of birth to the study respondents which provides some evidence for representativeness of the sample. There was a statistically significant difference between country of birth for mothers in this study compared to Victorian mothers in 2008. One possible explanation for this difference could be that the particular LGAs that were randomly selected for the survey differed in regards to mothers’ country of birth than when compared with the overall Victorian population. It is also possible that there was a difference in the return of the questionnaire based on mothers’ country of birth. There was insufficient information on non-responders to determine whether this has occurred.

A further limitation in this study was that participants were not asked when they were vaccinated relative to the birth of their child. As the questionnaire was administered approximately six months after the birth of their child, parents may not have been vaccinated in the initial two month period at which infants are most vulnerable prior to receiving their first dose of pertussis-containing vaccine at two months of age. Anecdotal information provided by parents in the comments section of the questionnaire indicated that some parents were vaccinated at the time of their child’s two month vaccinations. It is therefore possible that, among parents who were vaccinated at local councils or general practitioners, vaccination was received some time after their child’s birth: leaving their new baby vulnerable to infection, and thereby defeating the purpose of the program. Furthermore, the infant’s age at the time parents were surveyed may have impacted upon their responses to questions relating to their attitudes towards vaccination. It is plausible that a younger infant may be perceived by parents as more vulnerable to infection and hence a parent may be more supportive of vaccination. However, it is also plausible that the converse is true – an older infant might be considered more ‘robust’ to cope with a vaccination. The impact that the infant’s age had on parental attitudes towards vaccination was not able to be assessed.

It is also important to note that minor methodological differences between participating LGAs may have introduced some biases to the findings reported. Specifically, the study period was extended by one month in two rural LGAs in order to recruit sufficient study participants, although there was no change in the way the program was advertised or delivered during this time. Two potential participants in one rural shire were excluded due to incomplete names being provided by the LGA. One metropolitan LGA chose to post the questionnaires to participants directly, but unlike other LGAs, the introductory letters were not personalised and may account for a response rate of 30% for that LGA, which was the lowest response rate of participating metropolitan LGAs.

It is important to consider that the Victorian Government Department of Health initiated the free parental pertussis booster program in mid-2009 in response to the rising incidence of pertussis. The program ceased in Victoria on 30 June 2012 due to declining numbers of notified pertussis cases as well as limited evidence of the effectiveness of cocooning and doubts as to the cost effectiveness of the program. Similar cocooning programs introduced by other Australian jurisdictions, including South Australia, the Australian Capital Territory, Western Australia, and Queensland also ceased around the same time as Victoria, citing lack of evidence of effectiveness. A narrowed program targeting mothers in maternity hospitals continues in New South Wales [16]. Results of a case control study examining vaccine effectiveness of a similar cocooning program in New South Wales indicated that maternal vaccination was associated with a lower risk of pertussis among unimmunised infants (unadjusted OR 0.49; 95% C.I. 0.32-0.76) [17]. However, the study included mothers who had been vaccinated prior to the birth of their child, and as such, passive transfer of maternal antibodies may have contributed to the protective effect seen.

Although many countries around the world promote the cocooning strategy, the World Health Organization statement on pertussis vaccines states that “there is insufficient evidence to include this strategy in national immunisation programs” [18]. Several submissions to the Australian Pharmaceutical Benefits Advisory Committee for the vaccine to be included on the National Immunisation Program for parents have been rejected [19]. The Australian Government Department of Health and Ageing has since recommended that with the current absence of definitive evidence as to the effectiveness of the cocooning strategy at population level, practitioners should advise parents and other carers of infants less than six months of age to consider the potential benefits to themselves and their family of boosting their pertussis immunity, and that pertussis vaccine is available on prescription for parents and other carers who choose to receive it [20].

## Conclusions

This study found 70% of mothers and 53% of fathers were vaccinated for pertussis following their child’s birth (adjusted proportions of 68% and 49% for mothers and fathers, respectively). Whilst there is continued uncertainty regarding the effectiveness of cocooning as a strategy to reduce transmission of pertussis and subsequent morbidity and mortality to vulnerable infants, several factors were identified in this study that may encourage vaccine uptake should similar programs be implemented in future. These include stronger, specifically targeted communication messages, particularly relating to the susceptibility of adults to pertussis and the potential of pertussis to cause severe disease in infants. Further promotion of the widespread options and availability of vaccine providers may increase uptake for those who indicated time as a barrier to vaccination.

## Abbreviations

AUD: Australian dollar; CI: Confidence interval; dTpa: Diphtheria, tetanus and acellular pertussis vaccine; GP: General practitioner; LGA: Local government area; MCH: Maternal and child health; OR: Odds ratio; RR: Relative risk.

## Competing interests

The authors declare that they have no competing interests.

## Authors’ contributions

ED designed the study protocol, prepared the research and ethics applications, coordinated questionnaire design, administered the survey (including randomisation of participants, and conducting the data entry and analysis components) and coordinated the writing of the manuscript. JF contributed to questionnaire design, interpretation of data and significant drafting of the manuscript. SR assisted in research concept and study protocol development, garnered support for the research and contributed to the manuscript. LF identified important background material for the study protocol and manuscript, provided coordination with Maternal and Child Health Programs to identify participants and assisted in the drafting of the manuscript. HV contributed to the study design, questionnaire design, data analysis and drafting of the manuscript. All authors have read and approved the final manuscript.

## Pre-publication history

The pre-publication history for this paper can be accessed here:

http://www.biomedcentral.com/1471-2458/13/676/prepub
